# Serum free IgE guided dose reduction of omalizumab: a case report

**DOI:** 10.1186/s13223-017-0211-z

**Published:** 2017-08-31

**Authors:** Yasuhiro Gon, Reiko Ito, Shuichiro Maruoka, Kenji Mizumura, Yutaka Kozu, Hisato Hiranuma, Yuko Iida, Sotaro Shikano, Shu Hashimoto

**Affiliations:** Division of Respiratory Medicine, Nihon University School of Medicine, 30-1 Ohyaguchi-Kamicho, Itabashiku, Tokyo 173-8610 Japan

**Keywords:** Bronchial asthma, Omalizumab, IgE

## Abstract

**Background:**

Omalizumab is a human IgG1 antibody against IgE used as a therapy for sever asthmatic patients with asthma. According to the guidelines of the Global Initiative for Asthma, omalizumab is an add-on drug at treatment step 5 that is used for severe asthma patients who are allergic to perennial allergens. The effects of omalizumab for severe asthma therapy have been validated in multiple clinical studies. However, the long-term effects of omalizumab on IgE production and possibility of resetting of administration dose of omalizumab remain unknown.

**Case Presentation:**

The serum total and free IgE levels were measured over time in a 63-year-old female patient with allergic asthma who was administered 375 mg omalizumab biweekly for 36 months. Her symptoms did not worsen and clinical course remained favorable after reducing the dose to 375 mg per month. The serum free IgE levels temporarily increased following a dose reduction of omalizumab. The serum free IgE trough level temporarily increased at 4 weeks after capable to reduce the dosage; however, thereafter, the serum free IgE level decreased to desired levels (below 30 ng/mL).

**Conclusions:**

The present case shows the possibility of reducing the dose following the long-term use of omalizumab. Considering the high medical cost of omalizumab, the dose reduction may be a viable option. It may be useful to measure the serum free IgE level to appropriately identify patients in whom the dose can be reduced, and to carefully monitor the clinical course.

## To the editor

Omalizumab is a human IgG1 antibody against IgE used to treat patients with severe allergic asthma with symptoms that are poorly controlled with high doses of inhaled steroids. Numerous clinical studies have demonstrated the effectiveness of omalizumab therapy on intractable atopic asthma, and this has become one of the few available treatment modalities. The goal of omalizumab therapy is to achieve free IgE (unbound IgE that can bind to IgE receptors) of ≤25 ng/mL [1]; however, it is impossible to distinguish between free IgE and IgE-omalizumab complexes using conventional measurements of serum total IgE. Conventionally, the serum free IgE levels could not be assessed after administering omalizumab [2].

To overcome this issue, we previously developed ELISA with solid-phase soluble FcεRI to allow for the measurement of serum free IgE levels in patients undergoing omalizumab treatment [3]. Here we report the case of a patient who was able to reduce the dose of omalizumab supported by the measurement of the serum free IgE levels.

## Case

**Fig. 1:**
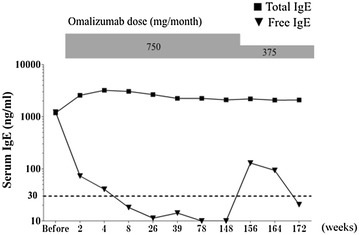
Changes in serum free and total IgE levels over time before and after omalizumab administration. Changes in serum free IgE (*Black up*-*pointing triangle*) and total IgE (*Black square*) levels are shown before commencing omalizumab (−1 week) to 172 weeks after beginning administration. The *longitudinal axis* represents the logarithmic expression of serum IgE levels

A 63-year-old female patient developed bronchial asthma at approximately 5 years of age. Her asthma did not spontaneously resolve and she subsequently suffered from regular asthma attacks. Specific IgE testing was positive for house dust, *Dermatophagoides pteronyssinus*, Chironomid midges, and negative for *Aspergillus fumigatus*, *Candida albicans*, *Penicillium notatum* and other common allergens. Despite the treatment with the combination of budesonide 1280 μg and formoterol 36 μg per day, a leukotriene receptor antagonist, and low-dose theophylline, she experienced exacerbations several times. The asthma control test (ACT) score was 18, and %FVC 86.7%, FEV1.0 0.88 mL, %FEV1 46.1% at 2 month before omalizumab therapy which initiated the year at in September 2013. Table 1 presents the term-course of her clinical data. She has a height of 157 cm and a weight of 66 kg, and her serum total IgE level was 580 IU/mL. Based on the omalizumab dose table, she was started on a biweekly administration of 375 mg omalizumab. At week 16, we conducted an evaluation to assess whether the drug should be continued. We observed an improvement in the patient’s subjective symptoms and morning peak expiratory flow levels. The level of airway obstruction had improved according to the respiratory function tests, and because there were no exacerbations within 16 weeks, we decided to continue omalizumab administration. Because there were no clear aggravations, we continued treatment with omalizumab at the same dose for 36 months. We measured her serum free IgE levels at 2, 4, 8, and 26 weeks after initiating omalizumab and found that the serum free IgE levels were extremely low at 72.8, 40.50, 18.1, and 11.3 ng/dL, respectively (Fig. 1). Because her symptoms had stabilized after initiating omalizumab with no exacerbations, she inquired whether it was possible to reduce the frequency of administration from once every 2 weeks to once every 4 weeks. Her ACT scores in the past 6 months were 23–25, her morning peak expiratory flow was stable, and there were no exacerbations in the past year; hence, we accepted her request and reduced the frequency of administration from biweekly to monthly without changing the dose of 375 mg omalizumab per administration beginning from August 2016. Therefore, the dosage was reduced by half. The serum free IgE trough level transiently increased to 129.3 ng/mL at 4 weeks after reducing the dosage; however, thereafter, the serum free IgE level decreased to 93.6 ng/mL within 4 weeks and gradually to 20.6 ng/mL, which is well below the desired level, 30 ng/mL (Fig. 1). She experienced no exacerbations during this period, and her ACT score remained at 25. FEV1 measured via pulmonary function testing almost unchanged after reduction of omalizumab (Fig. 1). Moreover, her asthma was controlled over the 9-month period since the dosage was reduced.

**Table 1:** Term-course of the patient’s clinical data before and after treatment of omalizumab

Date		Month-20xx							
		May-13	Jul-13	Sep-13	Nov-13	Jan-16	Jul-16	Sep-16	Jan-17
		Before omalizumab			Omalizumab 750 mg/month			Omalizumab 375 mg/month	
Blood test									
White blood cell no. (×10^3^)			6.4		6.2		6.8	6.3	7.9
Neutrophil no. (×10^3^)			3.9		4		5.1	4.5	6.15
Eosinophil (no.)			755		241		122	227	0.189
(%)			11.8		3.9		1.8	3.6	2.40%
Lung function test									
FVC (L)		1.93	2.13	2.15	2.28	2.27	2.58	2.26	2.47
%FVC (%)		78.1	85.9	86.7	92.3	91.9	105.7	92.6	102.5
FEV1.0 (L)		0.9	0.95	0.88	1.15	1.11	1.36	1.1	1.22
%FEV1.0 (% predicted)		44.3	46.1	42.7	56.7	54.7	68.5	55.6	63.5
FEV1.0/FVC (%)		40.9	42	37.1	47.9	48.5	51.1	44.9	48.2
FeNO									
(bbp)			40			18	21	20	20
Symptom score									
ACT			18		23	23	25	25	25

Asthma control test (ACT) assesses the frequency of shortness of breath and general asthma symptoms, use of rescue medications, the effect of asthma on daily functioning, and overall self-assessment of asthma control. ACT score 25 points indicates complete control of asthma, under 18 points indicates poor-controlled asthma 10)

## Discussion

At present, free IgE and IgE-omalizumab complexes cannot be distinguished using current clinical methods for measuring serum total IgE levels, like ImmunoCAP (Thermo Fisher Scientific, Uppsala, Sweden); thus, serum total IgE levels appear to increase following the administration of omalizumab [3, 4]. This is because of the 2.4-day half-life of human serum IgE compared to the 20-day half-life of IgE, which is longer because of IgE binding to the IgG1 antibody omalizumab.

Lowe et al. [4] have used mathematical models to predict that long-term omalizumab treatment may lower IgE production levels in their study of pharmacokinetic data from past clinical trials. According to the assessment using this model, the IgE production level is inhibited to a greater extent as the duration of omalizumab administration increases [4]. A prospective study measuring the free IgE level of 30 patients undergoing omalizumab therapy over time, using the method of free IgE measurement that we developed, demonstrated that free IgE levels 1 year after initiating administration had gradually decreased [4]. Furthermore, the speed at which the free IgE level decreases after initiating omalizumab has been demonstrated to have a possible association with the reactivity to omalizumab therapy [5]. In the present case, the total IgE levels also gradually declined beginning at 4 weeks. I suggested that the IgE production levels were observed to gradually decrease after initiating omalizumab administration, but it is difficult to fully grasp the quantitative relationship because the total IgE level temporarily increases after initiating omalizumab (Fig. 1).

While the mechanism underlying the decrease in the serum total IgE level due to the continuation of omalizumab therapy remains unknown, there are several possible explanations. First, decrease of free IgE levels by omalizumab can lead to down regulation of FcεRI on mast cell and dendritic cells [4]. Second, IgE production is promoted by Th2 cytokines and CD40 [6], omalizumab therapy may suppress the activation Th2 cells indirectly, thereby lowering IgE production. Third, the decreased expression of low-affinity IgE receptors on dendritic cells is also suggested to be potentially involved in decreasing IgE [4]. Third, the decreased expression of CD23/FcεRII, one of the low-affinity IgE receptors, may also be involved in suppressing IgE production. CD23/FcεRII is known to play an important role in maintaining IgE homeostasis. Past clinical research on the anti-human CD23/FcεRII monoclonal antibody lumiliximab has demonstrated that the inhibition of CD23/FcεRII controls the human serum IgE level [7]. The binding of CD23/FcεRII to IgE is reportedly inhibited by omalizumab bound to IgE [8]. Moreover, sCD23/FcεRII expression is reported to increase in response to inflammation [9]; thus, it is also possible that the improvement in allergic inflammation lowers soluble CD23/FcεRII, which then decreases the induction of IgE production via CD23/FcεRII.

The serum free IgE levels would be the most reliable marker reflect the IgE production status in the patients treated with omalizumab. One of the benefits of the long-term use of omalizumab is the improvement of patients’ atopic status by reducing IgE productivity. Moreover, if IgE production can be suppressed, it is possible to re-adjust and reduce the dosage.

## Conclusions

The present case shows the possibility of reducing the dose of omalizumab following its. Considering the high medical cost of omalizumab, reduction of the dose may be a viable option. In such a case, it is useful to measure the serum free IgE level to appropriately identify patients in whom the dose can be reduced. Because there are patients similar to the present study in whom serum free IgE levels temporarily increase following a dose reduction of omalizumab, it is recommended that their clinical courses be carefully monitored by observing their serum free IgE levels.
